# An Observational Study of Physical Activity in Parks in Asian and Pacific Islander Communities in Urban Honolulu, Hawaii, 2009

**Published:** 2011-08-15

**Authors:** Jane J. Chung-Do, Elise Davis, Stephanie Lee, Yuka Jokura, Lehua Choy, Jay E. Maddock

**Affiliations:** Office of Public Health Studies, University of Hawai'i at Mānoa; University of Hawai'i at Mānoa, Honolulu, Hawaii; University of Hawai'i at Mānoa, Honolulu, Hawaii; University of Hawai'i at Mānoa, Honolulu, Hawaii; University of Hawai'i at Mānoa, Honolulu, Hawaii; University of Hawai'i at Mānoa, Honolulu, Hawaii

## Abstract

**Introduction:**

Research on park use among Asians and Pacific Islanders is limited. This study examined use and conditions of 6 urban parks, varying in size, location, and neighborhood income level, in predominantly Asian and Pacific Islander communities in Honolulu, Hawaii. Sociodemographic predictors of park use were also identified.

**Methods:**

Observations were conducted from June through October 2009. Raters used the System for Observing Play and Recreation in Communities to count the number of people in predesignated zones and to code their physical activity level as sedentary, moderate, or vigorous. Raters coded park conditions on the basis of accessibility and usability, whether equipment and supervision were provided, and whether organized activities were occurring. Differences associated with sex and age of park users and income level of the neighborhood were examined by using χ² and logistic regression.

**Results:**

Raters observed 6,477 park users, most of whom were men. Approximately 60% of users were sedentary, 26% were engaged in moderate activities, and 14% performed vigorous activities. Women and girls were less active than men and boys. More users were present in the evenings, but morning users were more active. Although park users in low-income neighborhoods were more active than users in high-income neighborhoods, fewer people used the low-income parks. Most parks were accessible and usable but few provided equipment and supervision. Organized activities were rarely observed.

**Conclusion:**

More efforts should be made to promote parks as a physical activity resource in Asian and Pacific Islander communities, particularly for women, girls, and low-income residents. More research should be conducted to identify barriers and facilitators to park use, especially among underrepresented populations.

## Introduction

Although physical inactivity is a major public health issue in the United States, few studies have been conducted with Asians and Pacific Islander populations. Approximately 12 million Asian and Pacific Islanders live in the United States, accounting for approximately 5% of the total US population (1). The low proportion of Asians and Pacific Islanders has resulted in limited research with these populations, although a lower proportion of Asians and Pacific Islanders (38.6%) are physically active compared with the national average (45.8%) (2). Because physical inactivity contributes to poor health outcomes, health disparities exist among Asian and Pacific Islander populations. In Hawaii, where two-thirds of the state population is of Asian and Pacific Islander ancestry (3), Native Hawaiians and Filipinos are twice as likely as whites to report that their doctor has told them that they have diabetes (4).

Public parks play an important role in supporting physical activity; almost 87% of the national population reports regular or occasional park use (5). Public parks promote physical activity and are usually free or inexpensive compared with private facilities. To evaluate parks as a physical activity resource, park use needs to be accurately assessed (6). However, most studies rely on self-reported measures, which may be biased (7). To address this limitation, a reliable and valid measure of physical activity in parks has been developed: the System for Observing Play and Recreation in Communities (SOPARC) (8,9). SOPARC is a validated direct observation tool for assessing park conditions and park use, such as physical activity. Although SOPARC has been tested with racially diverse samples (10), it has not been used in predominantly Asian and Pacific Islander communities. The objective of this study was to use SOPARC to assess park use and conditions in 6 parks in communities with large numbers of Asians and Pacific Islanders in urban Honolulu and to identify sociodemographic factors that predict park usage.

## Methods

### Study settings and park selection

Trained raters observed park users in 6 Honolulu parks. Three of the parks have been designated by the Honolulu Department of Parks and Recreation as community parks and the other 3 as district parks. District parks are typically larger than community parks and are designed to serve more people (Honolulu Department of Parks and Recreation, oral communication, July 13, 2010). The parks were selected for the study on the basis of diversity in size, location, and income level of the surrounding neighborhood. The racial/ethnic populations in the census tracts surrounding the parks ranged from 53% to 74% Asians (Cambodian, Chinese, Indian, Japanese, Korean, Malaysian, Pakistani, Filipino, Thai, or Vietnamese) and from 3% to 10% Pacific Islanders (Native Hawaiians, Guamanians/Chamorros, Samoans, or other Pacific Islanders) (11). Therefore, the sample had proportions of Asians higher than that of the state and proportions of Pacific Islanders generally less than that of the state. The selected parks represented income categories by median annual household income of the surrounding census tract. These were low income (<$30,000), medium income (≥$30,000 and <$50,000) and high income (≥$50,000).

### Measures and procedures

SOPARC is a validated direct observation tool for assessing park conditions and physical activity of park users through periodic momentary scans in predesignated zones. All raters completed extensive training to ensure reliability of observations, which included conducting practice observations at parks (8,9). Raters were required to continue practice observations until they achieved at least 70% for both criterion validity and interrater reliability. A total of 757 paired observations were analyzed to assess interrater reliability. Agreement for park condition codes was above 97% and exceeded acceptable levels for reliability (12). Cohen's κ was calculated to assess the interrater reliability for physical activity levels. The mean score for all observations in this study was 0.84, ranging from 0.44 (vigorous boys) to 0.95 (sedentary girls), which met acceptable reliability levels (13).

Before data collection, 2 project managers mapped out observation zones at each park so that all park users within each zone were visible to the raters. Zones included athletic courts, sports fields, picnic areas, playgrounds, and open spaces, and excluded indoor facilities and parking lots. Data collection occurred from June through October 2009. For each park, observations were collected on 3 weekdays and 2 weekend days during 4 periods: morning (7-9 AM), noon (11 AM-1 PM), afternoon (2-4 PM), and evening (5-7 PM). Two raters arrived at parks 20 minutes before starting observations to assess safety and to review zoning boundaries. Overcrowded zones were subdivided for more accurate counting. Raters used 1 observation form for each zone to record the time, assess conditions, and rate physical activity levels of park users in the zone. Zones were coded as 1) *accessible* if the zone was open to the public (eg, not locked or rented to others), 2) *usable* if the zone was acceptable for physical activity (eg, not excessively wet or windy), 3) *equipped* if equipment was provided by the park and was available in the zone (eg, removable balls or other equipment), 4) *supervised* if the zone was supervised by designated park or adjunct personnel, 5) *organized* if an organized physical activity was occurring in the zone (eg, team sporting event) and 6) *empty* if no people were present in the zone. If a zone was empty, raters marked "yes" for empty, completed the information for the park conditions, and then moved to the next zone.

Raters conducted an observation by scanning the zone from left to right at an even tempo; they performed 4 scans per zone, 1 each for girls, boys, women, and men. Park users who appeared to be age 12 years or younger were recorded as children. Raters coded physical activity as *sedentary* if the rate of energy expended was less than energy expended when walking (eg, lying down, sitting, standing), *moderate* if a transfer of weight from 1 foot to another occurred (eg, walking) or *vigorous* if the energy expended was greater than casual walking (eg, running, push-ups or pull-ups, throwing or swinging) based on the activities that park users were engaged in during the scan. To simultaneously track the number of park users engaged in sedentary, moderate, or vigorous activity, raters used a mechanical counter. Raters then transferred the total counts to the observation form, reset the counters, and proceeded to the next scan. Once all scans in a zone were completed, raters moved to the next zone or subzone. All procedures were approved by the University of Hawai'i at Mānoa institutional review board.

### Data analysis

We performed *χ*² tests to examine bivariate associations between sociodemographic factors and physical activity levels. Because the dependent variable, the level of physical activity, was categorical (ie, sedentary, moderate, and vigorous), multivariate logistic regression was conducted to calculate odds ratios and 95% confidence intervals. Moderate and vigorous categories were compared with the probability of being sedentary, which served as the reference category. All significance levels were set at *P* < .05. Data from the observation forms were entered in SPSS version 14.0 (SPSS, Inc, Chicago, Illinois), which was used to conduct all analyses.

## Results

### Park usage and conditions

We provide descriptive information of the 6 selected parks (Table 1). Raters observed 6,477 park users across the parks (Table 2). More men and boys were observed using parks than women and girls, and more adults used parks than children. The majority of the park users observed were men, and girls were the least likely to be observed in parks. More people were observed in parks in high-income neighborhoods compared with middle-income neighborhoods and low-income neighborhoods. Parks were significantly more often used in the evening compared with earlier in the day. Organized activities were observed in 2.7% of the zones. Overall, park zones were found to be accessible and usable but were rarely equipped or supervised.

### Physical activity

Of all the park users observed, 60.2% were sedentary, and 25.6% were engaged in moderate and 14.2% in vigorous activities (Table 3). Men and boys were slightly more likely to engage in vigorous activities than women and girls. Children were twice as likely to engage in vigorous physical activities as adults. Women were most likely to be sedentary, and boys were most likely to engage in vigorous physical activity. People using low-income neighborhood parks were more likely to be active (engaging in moderate or vigorous physical activity) than users in middle- and high-income neighborhood parks. Park users were more likely to be sedentary later in the day; most vigorous activities occurred in the morning. All findings were significant (*P* < .001).

### Multivariate results

The sex and age of park users, neighborhood income level, and time of day significantly predicted physical activity level in the logistic regression (Table 4). Being female lowered the odds of being classified as physically active, while being a child increased the odds. Park users in lower-income neighborhood parks were associated with higher odds of being classified as physically active. Park users in middle-income neighborhood parks were more likely to be physically active than park users in high-income parks. The odds of being physically active were 84% higher in the morning than in the evening.

## Discussion

Overall patterns of park usage observed in our study were similar to results of studies in white, African American, and Hispanic communities (8,10,14). More than two-thirds of park users observed in our study were engaging in sedentary activities (eg, sitting, standing, lying down), and only 14% of park users were observed performing vigorous physical activity (eg, running, basketball, tennis). McKenzie et al found a similar pattern: 66% of parks users were sedentary and 16% were engaged in vigorous activity (8). The prevalence of sedentary activities in public parks raises questions about how public parks can better support physical activity. A study of minority communities in Los Angeles found that physically active park users were most commonly observed playing sports such as basketball, soccer, and baseball (15). Providing, promoting, and maintaining facilities for these sports may increase physical activity levels in parks.

Similar to findings of other studies, men and boys were more likely to use parks and engage in vigorous activities than women and girls (10,15). This finding reflects national data that Asian and Pacific Islander women are less likely to be physically active than their male counterparts (2). Moreover, our study found that girls aged 12 years or younger were least likely to be observed using parks compared to boys, women, and men. Future studies should identify factors that will help develop public health interventions and park programs to increase the number of women and girls who engage in moderate or vigorous physical activities in parks (16). For example, promoting sports and recreational activities in park facilities, such as playgrounds, basketball courts, walking paths, and multipurpose rooms, has been shown to increase moderate or vigorous physical activity among 6th-grade girls (17). A study of 25 community parks found that females used paved trails and swimming pools the most frequently (18). Tester and Baker also found that programmatic changes, such as expanded hours and improved youth and family programs, increase park usage and physical activity levels, especially among girls (19). Further research needs to be done to identify park preferences of Asian and Pacific Islander women and girls.

Significantly more people used parks in high-income neighborhoods than in lower-income neighborhoods. In fact, high-income parks attracted more than 3 times the number of park users as low-income parks. Studies have found that residents in low-income neighborhoods are more likely to perceive their neighborhoods as being unsafe (15,20,21), which is a barrier to park use (22). Improving park safety features, such as providing adequate lighting and interventions that improve community safety, may attract more people to public parks. Tester and Baker found that interventions improving the amenities and features of parks led to a 5- to 9-fold increase in the number of park users and increased physical activity levels among both male and female park users (19).

In contrast to other studies (6,23), we found that park users in low-income areas were more likely to engage in vigorous physical activity than users in middle-income and high-income parks. This finding also conflicts with studies that suggest people of lower socioeconomic status tend to be less physically active (24). Because we did not directly measure income levels of park users, users of parks located in low-income areas may not be of lower socioeconomic status. However, most people tend to use parks that are near their residence (25). More research needs to be conducted to understand park usage in low-income neighborhoods.

On the basis of census data, we found that low-income parks, which were less likely to be used, were located in neighborhoods with higher percentages of Pacific Islanders. According to the Behavioral Risk Factor Surveillance System, 75% of Pacific Islanders are considered to be overweight or obese compared with 52% of whites and 46% of Japanese people (26). Samoan children have the highest prevalence of obesity of all major racial/ethnic groups (27). Therefore, improving parks to increase usage may be an effective intervention to encourage physical activity among Pacific Islanders. Because public parks were reported as the most common place of exercise among minority populations (15), interventions should integrate cultural considerations.

The park conditions observed were similar to observations made by McKenzie et al (8). Most parks were accessible and usable throughout the day. However, we found that parks rarely provided equipment for physical activities, aside from permanent fixtures, such as basketball hoops and tennis nets. Most areas were not formally supervised and few organized activities were observed. McKenzie et al (8) found that providing organized activities can increase physical activity levels in parks. Furthermore, having formal supervision may increase park usage of children by addressing safety concerns of parents (28).

One of the limitations of this study is that race/ethnicity of individual park users was not collected. The high proportions of mixed-ethnicity people in Hawaii posed a challenge for raters to accurately categorize park users' ethnicities. Therefore, we could not directly ascertain how race/ethnicity is related to park usage and physical activity. Second, because observations were conducted only in outdoor areas, only outdoor park users were observed. We were also unable to assess duration of users' physical activity because our observations were based on momentary time sampling. Finally, other factors that were not included in this study may influence park usage, such as park incivilities, safety and crime concerns, park proximity to community residents, and awareness of park locations (16). Nevertheless, this was the first known observational study to examine park usage in predominantly Asian and Pacific Islander communities.

As the fastest growing minority group in the United States, the Asian and Pacific Islander population is projected to reach 20 million by 2020. By 2050, 1 of 10 Americans will be of Asian or Pacific Islander descent (1). Increasing our knowledge of how parks contribute to physical activity levels in the Asian and Pacific Islander population is an integral part of creating communities that support healthy lifestyles. The use of SOPARC in combination with other research methods, such as surveys and focus groups, may improve understanding of the barriers and facilitators to park use. This understanding may provide opportunities for partnerships among public health practitioners, city planners, and program specialists to promote a healthier environment. Knowing the factors that affect park usage can allow planners to create parks that prevent and reduce obesity and related conditions.

## Figures and Tables

**Table 1:** Demographic Characteristics of Neighborhoods Surrounding 6 Selected Parks, Honolulu, Hawaii,^a^ 2009

**N**	**Families Below Poverty, %**	**Median Household Annual Income, $**	**No High School Diploma, %**	**Asian, %**	**Pacific Islander, %**
**Low-income park A, <$30,000**					
3,842	17.7	25,451	45.0	73.9	7.2
**Low-income park B, <$30,000**					
3,465	23.0	28,210	35.1	67.7	10.0
**Middle-income park A, ≥$30,000 to <$50,000**					
7,054	9.3	39,387	12.0	52.9	7.4
**Middle-income park B, ≥$30,000 to <$50,000**					
4,073	4.5	48,077	18.4	63.2	5.9
**High-income park A, ≥$50,000**					
4,659	5.2	68,710	8.1	52.8	4.7
**High-income park B, ≥$50,000**					
3,270	2.0	81,153	7.1	72.3	2.7

a Source: US Census Bureau for census tracts surrounding the selected parks, 2000.

**Table 2:** Frequencies of Park Use Among 6,477 Park Users and Park Conditions of 2,603 Zones in 6 Selected Parks, Honolulu, Hawaii,^a^ 2009

**User Variable**	Park Users, n (%)
**Time of park use**	
Morning (7-9 AM)	767 (11.9)
Noon (11 AM-1 PM)	1,332 (20.7)
Afternoon (2-4 PM)	1,591 (24.7)
Evening (5-7 PM)	2,744 (42.6)
**Sex**	
Female	2,338 (36.1)
Male	4,139 (63.9)
**Age**	
**Child (≤12 y)**	1,862 (28.7)
Girls	730 (11.3)
Boys	1,132 (17.5)
**Adult (>12 y)**	4,615 (71.3)
Women	1,608 (24.8)
Men	3,007 (46.4)
**Neighborhood income level**	
Low (<$30,000)	943 (14.6)
Middle (≥$30,000 and <$50,000)	2,330 (36.0)
High (≥$50,000)	3,204 (49.5)

**Conditions**	**Zones, No. (%)**

Equipment was provided by the park	3 (0.1)
Supervision provided by park personnel	37 (1.4)
Organized physical activity occurring	70 (2.7)
Usable for physical activity	2,571 (98.3)
Accessible to the public	2,591 (99.0)

a A zone is defined as an area of the park designated by project managers for the System for Observing Play and Recreation in Communities observations. Areas included athletic courts, sports fields, picnic areas, playgrounds, open spaces, and other areas within the park, excluding indoor facilities and parking lots.

**Table 3:** Bivariate Associations for Sociodemographic Factors, by Physical Activity Level, of 6,477 Park Users, Honolulu, Hawaii, 2009

Variables and Attribute Levels	Level of Physical Activity^a^			χ^2^	*P* Value

	Sedentary, %	Moderate, %	Vigorous, %		
**Total**	60.2	25.6	14.2	NA	NA
**Time of park use**					
Morning (7-9 AM)	45.9	31.8	22.3	122.51	<.001
Noon (11 AM-1 PM)	59.3	30.2	10.5		
Afternoon (2-4 PM)	65.3	22.7	12.0		
Evening (5-7 PM)	61.6	23.3	15.1		
**Sex**					
Female	64.1	23.8	12.4	25.43	<.001
Male	58.0	26.5	15.4		
**Age**					
Children (≤12 y)	56.9	22.4	20.7	92.98	<.001
Adults (>12 y)	61.6	26.8	11.6		
**Park users, grouped by sex and age**					
Girls	59.2	20.5	20.3	125.80	<.001
Boys	55.4	23.6	21.0		
Women	66.4	25.3	8.3		
Men	59.0	27.6	13.3		
**Neighborhood annual income level, $**					
Low (<30,000)	49.5	34.3	16.2	68.40	<.001
Middle (≥30,000 to <50,000)	60.0	24.4	15.6		
High (≥50,000)	63.5	23.8	12.6		

Abbreviation: NA, not applicable.

a Sedentary defined as a rate of energy less than that expended when walking; moderate defined as transfer of weight from 1 foot to another; vigorous defined as a rate of energy expended greater than casual walking.

**Table 4:** Multinomial Logistic Regression for Sociodemographic Factors, by Physical Activity Level, of 6,477 Park Users, Honolulu, Hawaii, 2009

Variables and Attribute Levels	Sedentary Activity^a ^vs Physically Active^b^	

	Exp [β], (95% CI)	*P* Value
**Time of park use**		
Morning (7-9 AM)	1.84 (1.56-2.17)	<.001
Noon (11 AM-1 PM)	1.18 (1.03-1.35)	.02
Afternoon (2-4 PM)	0.81 (0.71-0.92)	.001
Evening (5-7 PM)	1 [Reference]	
**Sex**		
Female	0.75 (0.68-0.84)	<.001
Male	1 [Reference]	
**Age**		
Child (≤12 y)	1.32 (1.18-1.48)	<.001
Adult (>12 y)	1 [Reference]	
**Neighborhood annual income level, $**		
Low (<30,000)	1.75 (1.50-2.03)	<.001
Middle (≥30,000 to <50,000)	1.19 (1.06-1.33)	.002
High (≥50,000)	1 [Reference]	

Abbreviations: CI, confidence interval; Exp [β], odds ratio.

a Sedentary activity served as the reference activity and was defined as a rate of energy less than that expended when walking (eg, lying down, sitting, standing).

b Physically active was defined as moderate if weight was transferred from 1 foot to another (eg, walking) or vigorous if the energy expended was greater than for casual walking (eg, running, push-ups or pull-ups, throwing or swinging).

## References

[1] Environmental Protection Agency. Asian American and Pacific Islander primer.

[2] Centers for Disease Control and Prevention (2004). Physical activity among Asians and Native Hawaiian or other Pacific Islanders — 50 states and the District of Columbia, 2001-2003. MMWR Morb Mortal Wkly Rep.

[3] United States Census Bureau. State and county quickfacts: state of Hawaii.

[4] Hawaii Department of Health. Have you ever been told by doctor that you have diabetes? State of Hawaii BRFSS 2008.

[5] National Survey on Recreation and the Environment (NSRE2000). Summary report no. 4. Americans' lifestyles, 2000.

[6] Floyd M, Spengler JO, Maddock JE Assessing the population impact of public parks on physical activity: an analysis of energy expenditure and neighborhood parks. Paper presented at: Active Living Research Conference.

[7] Adams S, Matthews C, Ebbelling CB, Moore C, Cunningham J, Fulton J, Hebert J (2005). The effect of social desirability and social approval on self-reports of physical activity. Am J Epidemiol.

[8] McKenzie TL, Cohen DA, Sehgal A, Williamson S, Golinelli D (2006). System for Observing Play and Recreation in Communities (SOPARC): reliability and feasibility measures. J Phys Act Health.

[9] Active Living Research. Building the evidence to prevent childhood obesity and support active communities.

[10] Floyd MF, Spengler JO, Maddock JE, Gobster PH, Suau LJ (2008). Park-based physical activity in diverse communities of two US cities. Am J Prev Med.

[11] Humes KR, Jones NA, Ramirez RR Overview of race and Hispanic origin: 2010. 2010 Census Briefs. US Census Bureau.

[12] Riffe D, Stephen L, Frederick GF (1998). Analyzing media messages: using quantitative content analysis in research.

[13] Landis JR, Koch GG (1977). The measurement of observer agreement for categorical data. Biometrics.

[14] Floyd MF, Spengler JO, Maddock JE, Gobster PH, Suau L (2008). Environmental and social correlates of physical activity in neighborhood parks: an observational study in Tampa and Chicago.

[15] Cohen D, McKenzie T, Sehgal A, Williamson S, Golinelli D, Lurie N (2007). Contribution of public parks to physical activity. Am J Public Health.

[16] Jilcott SB, Evenson KR, Laraia BA, Ammerman AS (2007). Association between physical activity and proximity to physical activity resources among low-income, midlife women. Prev Chronic Dis.

[17] Kaczynski A Factors influencing awareness and use of parks for physical activity. Presented at: Kansas Department of Health and Environment Summit December 1, 2009 Topeka, Kansas Kaczynski A. Factors influencing awareness and use of parks for physical activity. Presented at: Kansas Department of Health and Environment Summit; December 1, 2009; Topeka, Kansas.

[18] Reed JA, Arant CA, Wells P, Stevens K, Hagen S, Harring HA (2008). A descriptive examination of the most frequently used activity settings in 25 community parks using direct observation. J Phys Act Health.

[19] Tester J, Baker R (2009). Making the playfields even: evaluating the impact of an environmental intervention on park use and physical activity. Prev Med.

[20] Wilson D, Kirtland K, Ainsworth B, Addy C (2004). Socioeconomic status and perceptions of access and safety for physical activity. Ann Behav Med.

[21] Scott D, Munson W (1994). Perceived constraints to park usage among individuals with low incomes. J Park Recreat Admi.

[22] Grzywacz JG, Marks NF (2001). Social inequalities and exercise during adulthood: toward an ecological perspective. J Health Soc Behav.

[23] Parks SE, Housemann RA, Brownson RC (2003). Differential correlates of physical activity in urban and rural adults of various socioeconomic backgrounds in the United States. J Epidemiol Community Health.

[24] Evans GW, Katrowitz E (2002). Socioeconomic status and health: the potential role of environmental risk exposure. Annu Rev Public Health.

[25] Giles-Corti B, Donovan RJ (2002). The relative influence of individual, social and physical environment determinants of physical activity. Soc Sci Med.

[26] Davis J, Busch J, Hammat Z, Novotny R, Harrigan R, Grandinetti A, Easa D (2004). The relationship between ethnicity and obesity in Asian and Pacific Islander populations: a literature review. Ethn Dis.

[27] Baruffi G, Hardy CJ, Waslien CI, Uyehara SJ, Krupitsky D (2004). Ethnic differences in the prevalence of overweight among young children in Hawaii. J Am Diet Assoc.

[28] Veitch J, Bagley S, Ball K, Salmon J (2006). Where do children usually play? A qualitative study of parents' perceptions of influences on children's active free-play. Health Place.

